# First Report on Detection and Complete Genomic Analysis of a Novel CRESS DNA Virus from Sea Turtles

**DOI:** 10.3390/pathogens12040601

**Published:** 2023-04-15

**Authors:** Kerry Gainor, Kimberly M. Stewart, Angela Picknell, Morgan Russ, Noah Makela, Kierra Watson, Diana M. Mancuso, Yashpal Singh Malik, Souvik Ghosh

**Affiliations:** 1 Department of Biomedical Sciences, Ross University School of Veterinary Medicine, Basseterre P.O. Box 334, Saint Kitts and Nevis; kerrygainor@students.rossu.edu (K.G.); kstewart@rossvet.edu.kn (K.M.S.); angie.skturtles@gmail.com (A.P.); morganruss@students.rossu.edu (M.R.); noahmakela@students.rossu.edu (N.M.); kierrakwatson@gmail.com (K.W.); dianamancuso@students.rossu.edu (D.M.M.); 2 St. Kitts Sea Turtle Monitoring Network, Basseterre P.O. Box 2298, Saint Kitts and Nevis; 3 College of Animal Biotechnology, Guru Angad Dev Veterinary and Animal Science University, Ludhiana 141012, India; malikyps@gmail.com

**Keywords:** complete genome analysis, *Cressdnaviricota*, novel CRESS DNA virus, sea turtle

## Abstract

To date, only a handful of viruses have been identified in sea turtles. Although eukaryotic circular Rep (replication initiation protein)-encoding single-stranded DNA (CRESS DNA) viruses have been reported from a wide variety of terrestrial species, and some of these viruses have been associated with clinical conditions in certain animals, limited information is available on CRESS DNA viruses from marine life. The present study aimed to investigate the presence of CRESS DNA viruses in sea turtles. In the present study, two (samples T3 and T33) of the 34 cloacal samples from 31 sea turtles (found in ocean waters around the Caribbean Islands of St. Kitts and Nevis) tested positive for CRESS DNA viruses by a pan-*rep* nested PCR assay. The partial Rep sequence of T3 shared 75.78% of a deduced amino acid (aa) identity with that of a CRESS DNA virus (classified under family *Circoviridae*) from a mollusk. On the other hand, the complete genome (2428 bp) of T33 was determined by an inverse nested PCR assay. The genomic organization of T33 mirrored those of type II CRESS DNA viral genomes of cycloviruses, characterized by the putative “origin of replication” in the 5’-intergenic region, and the putative Capsid (Cap)- and Rep-encoding open reading frame on the virion-sense- and antisense-strand, respectively. The putative Rep (322 aa) of T33 retained the conserved “HUH endonuclease” and the “super 3 family helicase” domains and shared pairwise aa identities of ~57% with unclassified CRESS DNA viruses from benthic sediment and mollusks. Phylogenetically, the T33 Rep formed a distinct branch within an isolated cluster of unclassified CRESS DNA viruses. The putative Cap (370 aa) of T33 shared maximum pairwise aa identity of 30.51% with an unclassified CRESS DNA virus from a capybara. Except for a blood sample from T33 that tested negative for CRESS DNA viruses, other tissue samples were not available from the sea turtles. Therefore, we could not establish whether the T3 and T33 viral strains infected the sea turtles or were of dietary origin. To our knowledge, this is the first report on the detection of CRESS DNA viruses from sea turtles, adding yet another animal species to the rapidly expanding host range of these viruses. Complete genome analysis of T33 identified a novel, unclassified CRESS DNA virus, providing insights into the high genetic diversity between viruses within the phylum *Cressdnaviricota*. Considering that sea turtles are an at-risk species, extensive studies on virus discovery, surveillance, and pathogenesis in these marine animals are of the utmost importance.

## 1. Introduction

Sea turtles (Superfamily *Chelonioidea*) are migratory marine reptiles that primarily thrive in tropical and subtropical ocean/seas worldwide [1,2,3]. To date, at least seven species of sea turtles (Australian flat-back (*Natator depressa*), greens (*Chelonia mydas*), hawksbills (*Eretmochelys imbricata*), Kemp’s ridley (*Lepidochelys kempii*), leatherbacks (*Dermochelys coriacea*), loggerheads (*Caretta caretta*), and olive ridley (*Lepidochelys olivacea*)) have been formally recognized [1,2,3]. During the last 100 years, there has been a significant reduction in the global population of sea turtles, which has mostly been attributed to direct and indirect human activities [4]. The International Union for Conservation of Nature (IUCN) has listed six of the seven species of sea turtles as critically endangered, endangered, or vulnerable animals in the IUCN red list, whilst the seventh species (flatback) has been reported as “Data Deficient”, warranting extensive conservation efforts [5].

Infectious diseases are considered among the top five reasons for the extinction of terrestrial species [6]. On the other hand, limited information is available on various pathogens and pathogen-related morbidity and mortality in marine wildlife, including the sea turtle [4]. To date, only a handful of viruses have been identified in sea turtles [4,7,8,9]. Cheloniid herpesviruses (Cheloniid herpesvirus-1, -5, and -6) are the most studied viruses and have been linked to clinical diseases (Grey patch disease, fibropapillomas, and lung-eye-trachea disease, respectively) in sea turtles with severe outcomes in stressed/immunosuppressed animals [4,7,8,9]. Loggerhead genital-respiratory herpesvirus (LGRV) and loggerhead orocutaneous herpesvirus (LOCV) have been associated with oral, respiratory, genital, and cutaneous lesions in wild loggerhead turtles [10]. Papillomaviruses (*Chelonia mydas* papillomavirus (CmPV-1) and *Caretta caretta* papilloma virus (CcPV-1) associated with proliferative dermatitis) [11,12,13], a novel poxvirus (Cheloniid poxvirus 1) [14], retrovirus [15], betanodavirus [16], and tornovirus [17] have been sporadically detected in sea turtles.

The recently established phylum *Cressdnaviricota* consists of a large number of eukaryotic, circular, single-stranded DNA viruses that encode a characteristic and conserve two-domain (the N-terminal HUH endonuclease domain and the C-terminal superfamily 3 helicase (S3H) domain) replication initiation proteins (Rep) [18]. Informally, these viruses are known as “circular Rep-encoding single-stranded (CRESS) DNA viruses” [18]. Based on phylogenetic analysis of Rep protein sequences, CRESS DNA viruses have been classified into at least seven established families (*Bacilladnaviridae*, *Circoviridae*, *Geminiviridae*, *Genomoviridae*, *Nanoviridae*, *Redondoviridae*, and *Smacoviridae*), six well-defined groups of unclassified CRESS DNA viruses (designated as CRESSV1-6), and several other unclassified CRESS DNA viruses [18]. On the other hand, the capsid (Cap) proteins of CRESS DNA viruses are not orthologous and have been proposed to be derived from RNA viruses [18,19,20,21]. CRESS DNA viruses are a diverse and expanding group of small viruses that have been detected in a wide variety of living forms (vertebrates, invertebrates, plants, fungi, and algae) and in environmental samples [18,21,22]. Some of the CRESS DNA viruses (circoviruses in animals, geminiviruses, and nanoviruses in plants) have been linked to symptomatic infections, whilst the disease-causing potential and even the true host/s-of-origin remain to be properly elucidated for many CRESS DNA viruses [18,22].

Although CRESS DNA viruses are ubiquitous and have been reported from a wide variety of terrestrial species, limited information is available on these viruses from marine life [21,22,23,24,25,26,27,28]. To date, there are no reports on CRESS DNA viruses in sea turtles. In the present study, for the first time, we identified two CRESS DNA viral strains in cloacal samples from sea turtles. Complete genomic analysis of one of the sea turtles associated with CRESS DNA viral strains revealed a novel, unclassified CRESS DNA virus.

## 2. Materials and Methods

### 2.1. Sampling

During June 2021–August 2022, a total of 34 cloacal samples were obtained from 31 sea turtles (3 turtles were sampled twice, in 2021 and in 2022) by members of the St. Kitts Sea Turtle Monitoring Network (SKSTMN), a nonprofit organization dedicated to the monitoring and rehabilitation of sea turtles in coastal waters around the Caribbean Islands of St. Kitts and Nevis [29]. These samples were collected to create a biobank for future research activities and became available for the present study. Information (sea turtle species, sampling site, month and year of collection, and presence of lesions/epibiota) related to the sea turtle cloacal samples is shown in Supplementary Table S1. Of the 31 sampled turtles, 29 animals (20 greens and 9 hawksbill sea turtles, all apparently healthy juveniles with mild to moderate epibiota loads) were captured via snorkel from coastal waters, were immediately brought to the nearest beach (where they were sampled), and were released after routine health assessments and tagging (if found untagged). Two of the juvenile sea turtles were found to have mild proliferative cutaneous lesions, consistent with the neoplastic conditions observed in fibropapillomatosis. On the other hand, the remaining 3 samples were collected from sea turtles (two subadult loggerheads (T32 and T33) and one juvenile hawksbill (T34)) that were undergoing post-injury rehabilitation at SKSTMN. A single blood sample (collected around the time (June 2021) of cloacal sampling) was also available from sea turtle T33.

In the present study, the sea turtles were sampled in accordance with the regulations set forth by the Institutional Animal Care and Use Committee (IACUC) of the Ross University School of Veterinary Medicine (RUSVM), St. Kitts and Nevis, under approved IACUC protocol # 20.10.25 and the St. Kitts Department of Marine Resources. Briefly, the sea turtles were manually restrained, and a sterile cloacal swab (MicroTest™ M4RT, Thermo Fisher Scientific Inc., Waltham, MA, USA) was inserted approximately 6 cm into the cloaca and rotated five times. The cloacal swab was transferred into a sterile tube containing viral transport medium (MicroTest™ M4RT, Thermo Fisher Scientific Inc., Waltham, MA, USA). The blood sample was collected from the dorsal cervical vessel of sea turtle T33 using a 22-gauge 2.5 cm sterile needle (Nipro Medical, Bridgewater, NJ, USA) attached to a syringe (pre-coated with sodium heparin) (Covidien Ltd., Dublin, Ireland) under aseptic conditions. The blood sample represented <0.1% of the body weight of T33. The samples were shifted to the research laboratory at RUSVM and stored at −80 °C until further analysis.

### 2.2. Amplification of Viral DNA

Viral DNA was extracted from the cloacal samples and the blood sample using the QIAamp DNA Mini Kit (Qiagen Sciences, Germantown, MD, USA) and the DNeasy Blood & Tissue Kit (Qiagen Sciences, Germantown, MD, USA), respectively, following the manufacturers’ instructions. The sea turtle samples were screened for the presence of CRESS DNA viruses by a broad-spectrum nested PCR assay that was shown to successfully amplify a short stretch (~400 bp) of the *rep* gene from diverse CRESS DNA viruses, as described previously [30,31,32]. Two pairs of primers, designed from the obtained ~400 nucleotide (nt) viral *rep* sequences, were employed in inverse nested PCRs to amplify the complete genomes of the sea turtle-associated CRESS DNA viruses. The screening and nested PCRs were performed using Platinum™ Taq DNA Polymerase (Invitrogen™, Thermo Fisher Scientific Corporation, Waltham, MA, USA) according to the manufacturer’s instructions, and included sterile water as the negative control in all reactions.

### 2.3. Nucleotide Sequencing

The PCR products were purified by a commercially available kit (Wizard^®^ SV Gel and PCR Clean-Up kit, Promega, Madison, WI, USA), following the manufacturer’s instructions. Nucleotide sequences were generated by Sanger sequencing using the ABI Prism Big Dye Terminator Cycle Sequencing Ready Reaction Kit (Applied Biosystems, Foster City, CA, USA) on an ABI 3730XL Genetic Analyzer (Applied Biosystems, Foster City, CA, USA). The viral genome was sequenced in both directions using the PCR primers and additional internal primers (designed from the obtained sequences). To confirm the sequencing results, the entire process (viral DNA extraction to sequencing) was repeated.

### 2.4. Sequence Analysis

The map of the sea turtle-associated CRESS DNA viral genome was created with the ‘Draw Custom Plasmid Map’ program (https://www.rf-cloning.org/savvy.php, accessed on 27 February 2023). The mFold program was employed to locate the putative stem-loop structure on the viral genome [33]. The putative Rep- and Cap- encoding open reading frames (ORFs) were identified using the ORF finder (https://www.ncbi.nlm.nih.gov/orffinder/, accessed on 25 February 2022). Homology searches for related CRESS DNA viral sequences were performed using the standard BLASTN and BLASTP program (Basic Local Alignment Search Tool, www.ncbi.nlm.nih.gov/blast, accessed on 25 February 2023). Pairwise sequence (%) identities (for the complete viral genome and the putative Rep and Cap) were determined using the MUSCLE algorithm embedded in the SDTv1.2 program, as described previously [31,32,34,35]. Phylogenetic analysis of the putative Rep was performed by the maximum likelihood (ML) method (rtREV + G + I + F amino acid (aa) model of substitution, 1000 bootstrap replicates) using the MEGA11 software (downloaded from https://www.megasoftware.net/ on 1 February 2023), as described previously [18,19].

### 2.5. GenBank Accession Numbers

The GenBank accession numbers for the sea turtle-associated CRESS DNA virus sequences (T3 and T33) determined in this study are OQ606817 and OQ606816, respectively.

## 3. Results and Discussion

**Figure 1:**
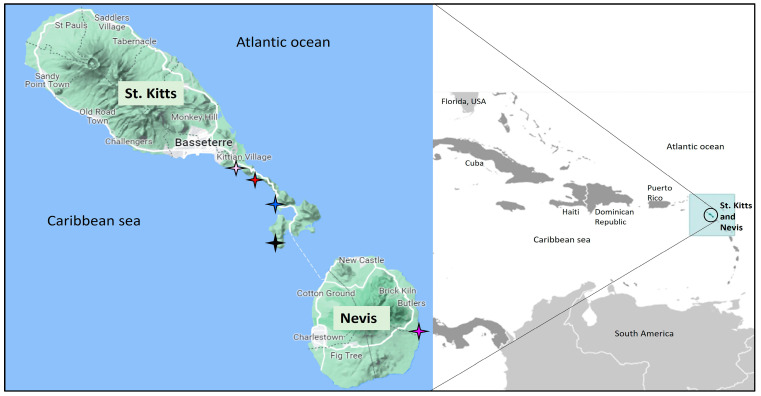
Geographical position of the Islands of St. Kitts and Nevis in the Caribbean region (**Right**). The map was adapted from https://www.cia.gov/library/publications/the-world-factbook (accessed on 1 April 2021). Map of St. Kitts and Nevis showing the locations (shown with colored stars) where the sea turtles were sampled or rescued after being stranded (**Left**). Black star: Shitten Bay; Blue star, Whitehouse Bay; Pink star, Timothy beach; Purple star, New River; Red star, South Friars beach. The map was adapted from https://www.google.com/maps (accessed on 2 February 2023).

The Caribbean Islands of St. Kitts and Nevis (combined area of 104 square miles, human population of ~50,000) are surrounded by the Caribbean Sea and the Atlantic Ocean (Figure 1) [36]. Juvenile and subadult hawksbill and green sea turtles have been observed in tropical waters around St. Kitts and Nevis throughout the year, with adults of these species as well as leatherbacks utilizing the beaches for seasonal nesting [29]. Loggerhead sea turtles have rarely been seen in the offshore waters surrounding St. Kitts and Nevis, with only three formal reports since the 1960s [29].

**Figure 2:**
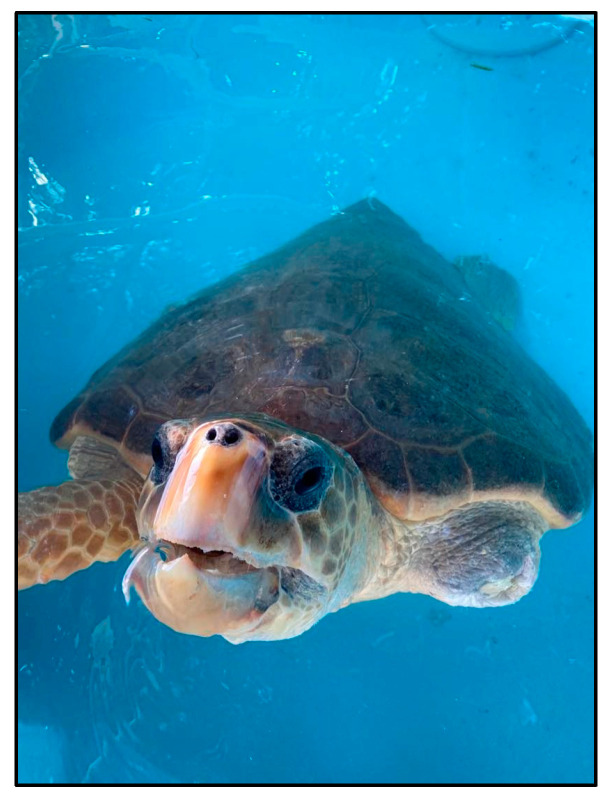
Sea turtle T33, also known as Kayden. The sea turtle-associated CRESS DNA virus T33 was detected in a cloacal sample from Kayden.

In the present study, two (samples T3 and T33) of the 34 cloacal samples from sea turtles yielded the expected ~400 bp amplicon with the CRESS DNA virus pan-*rep* nested PCR assay [30] (Supplementary Table S1). The single blood sample from sea turtle T33 tested negative for CRESS DNA viruses. The T3 cloacal sample was obtained from a juvenile hawksbill turtle at Whitehouse Bay, St. Kitts, in November of 2021. Other than a moderate load of barnacles and algae on the caudal portion of the carapace, sea turtle T3 appeared healthy. The turtle was sampled again (sample T20) from the same location on April 2022, as evidenced from the identification tag (Supplementary Table S1), and tested negative for CRESS DNA viral DNA. Sea turtle T33 (also known as Kayden, a subadult loggerhead) was found stranded at the mouth of New River Ghaut in Nevis and was rescued by SKSTMN in October of 2019 [37] (Figure 2). Kayden was emaciated and had sustained extensive injuries, requiring amputation of the left front flipper [37]. Following complete recovery, T33/Kayden was sampled and released in June of 2021 [37].

The PCR amplicons from samples T3 and T33 were sequenced to confirm the screening results. By BLASTN/P analysis, the partial *rep*/Rep sequence from T3 shared maximum nt and a deduced amino acid (aa) identity of 75.19% (query cover: 100%) and 75.78% (query cover: 100%), respectively, with that of CRESS DNA virus Lake Sarah-associated circular virus-14 isolate LSaCV-14-LSMU-2013 (classified under family *Circoviridae*, GenBank accession number KP153414) from a New Zealand freshwater mussel (*Echyridella menziesii*) [19,38]. On the other hand, the partial rep sequence from T33 shared maximum nt (73.49% identity, query cover: 32%) and a deduced aa (55.35% identity, query cover: 94%) identity with that of Bat circovirus ZS/China/2011 (GenBank accession number JF938128) and unclassified CRESS DNA virus Avon-Heathcote Estuary-associated circular virus 14 (KM874332) from a New Zealand little neck clam (*Austrovenus stutchburyi*) [23], respectively. The partial *rep*/Rep sequences of T3 and T33 shared pairwise nt and deduced aa identities of 56.91% and 41.26%, respectively, between themselves. The cloacal samples were also screened for the presence of adenoviral (AdV) DNA and picobirnavirus (PBV) RNA, as described previously [39,40]. However, none of the sea turtle samples from the study tested positive for AdV and PBV (data not shown).

Since CRESS DNA viruses contain a circular DNA molecule [18,22], we determined the complete genome of sea turtle-associated CRESS DNA virus T33 by an inverse nested PCR assay using primers designed from partial *rep* sequences (obtained during the screening process). On the other hand, a similar approach failed to amplify the whole genome of CRESS DNA virus T3, although T3 exhibited a strong amplification with the screening of the pan-*rep* nested PCR assay. The complete genome of CRESS DNA virus T33 was 2428 nt in size (GC content: 41.68%), which was comparable to those of CRESS DNA viral families *Genomoviridae* (2–2.4 kb), *Geminiviridae* (2.5–3 kb/segment), and *Smacoviridae* (2.3–2.9 kb), larger than *Circoviridae* (1.7–2.1 kb) and *Nanoviridae* (0.98–1.1 kb/segment), and smaller compared with *Bacilladnaviridae* (5.5–6 kb) and *Redondoviridae* (3–3.1 kb) [18]. BLASTN analysis of the complete genome of T33 was inconclusive (query cover: 18%), with nt sequence identities of 70.70–72.45% between a partial (~450 nt) stretch of the ~2.4 kb T33 sequence and cognate sequences of CRESS DNA viruses from minnow tissue (GenBank accession number MH617696) and mollusks (KM87432-35 and -46).

**Figure 3:**
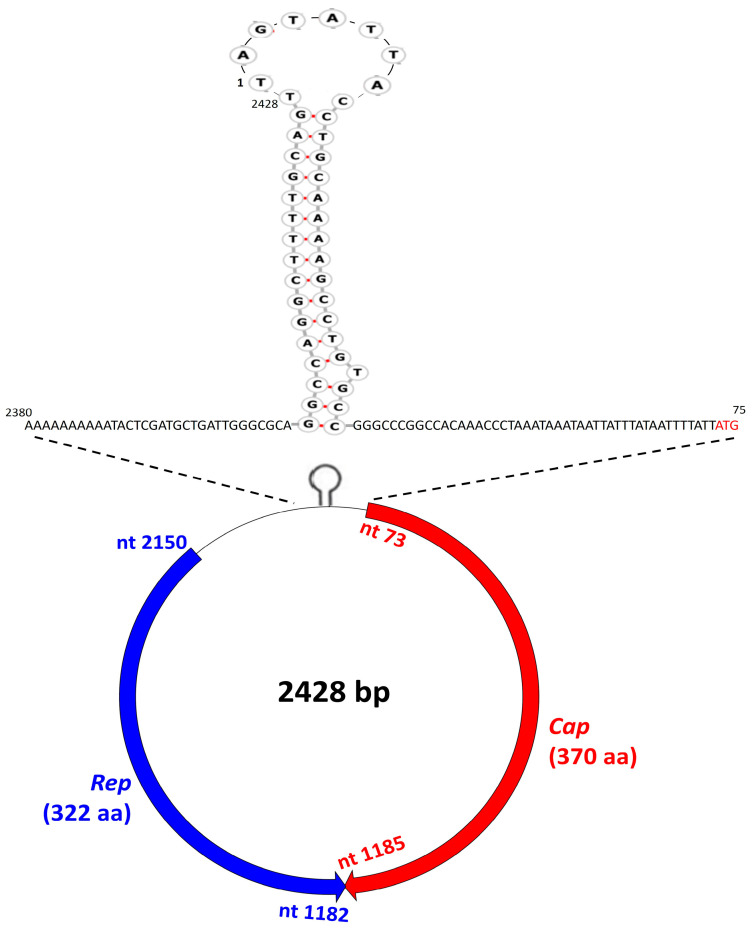
The genomic organization of sea turtle-associated CRESS DNA virus T33. The open reading frames encoding the putative Capsid (Cap) and Replication initiation protein (Rep) are shown with red and blue arrows, respectively. The 5’- intergenic region of T33 contained the putative origin of replication on the Cap-encoding strand, which was characterized by the presence of a nonanucleotide motif (TAGTATTAC) at the apex of a well-defined stem-loop structure. The initiation codon for the putative Cap of T33 is shown with red font. The sizes of the Rep and Cap are shown in parentheses. nt: nucleotide; aa: amino acid.

**Figure 4:**
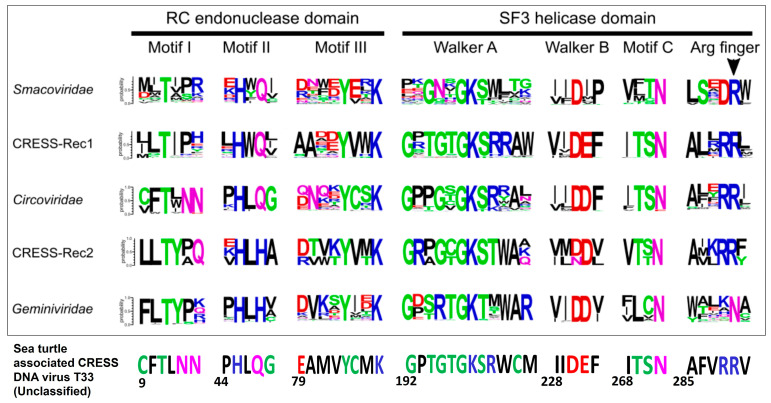
The rolling circle replication (motifs I–III) and superfamily 3 helicase (Walker-A and -B, motif C, and arginine finger motif) motifs in the putative replication initiation proteins (Rep) of sea turtle-associated CRESS DNA virus T33 and other representative CRESS DNA viruses. Acidic, basic, hydrophobic, neutral, and polar amino acid residues are shown with red, blue, black, purple, and green color font, respectively. The numbers below the motif sequences of T33 correspond to the positions of the amino acid residue in the T33 Rep protein. The boxed section in Figure 4 was adapted from Figure 2 of Kazlauskas et al. [19] (licensed for free sharing and adaptation under the Creative Commons Attribution 4.0 International license, CC BY 4.0).

Applying the standard genetic code (transl_table = 1) (https://www.ncbi.nlm.nih.gov/Taxonomy/Utils/wprintgc.cgi, accessed on 25 February 2023) with ‘ATG’ as the initiation codon, the T33 sequence was found to contain at least two inversely arranged putative ORFs with a 4 nt overlap at the 3’- end of respective ORFs (Figure 3). The putative ORF (nt 73–nt 1185) on the virion-sense (positive-sense) strand encoded a polypeptide (370 aa) that shared maximum homology with the capsid proteins of CRESS DNA viruses (Figure 3). On the other hand, the putative ORF (nt 2150-nt 1182) on the complementary (antisense) strand coded for a polypeptide (322 aa) that retained two conserved Rep domains (the HUH endonuclease and S3H domains) and shared maximum homology with the Rep of CRESS DNA viruses (Figure 3 and Figure 4). The 5’- intergenic region (the region between the two ORFs) of T33 contained the putative origin of replication (*ori*) on the Cap-encoding strand, which was characterized by the presence of a nonanucleotide motif (TAGTATTAC) at the apex of a well-defined stem-loop structure (Figure 3). The putative *ori* plays an important role in initiating viral genome replication and is conserved in CRESS DNA viruses [22]. Following the guidelines for analysis of CRESS DNA viral genomes [22,41], the first nt residue (T) of the putative nonanucleotide motif was assigned as “nt position one” in the T33 sequence (Figure 3). Taken together, the genomic organization of sea turtle-associated CRESS DNA virus T33 mirrored those of type II CRESS DNA viral genomes of cycloviruses [41] (Figure 3).

**Figure 5:**
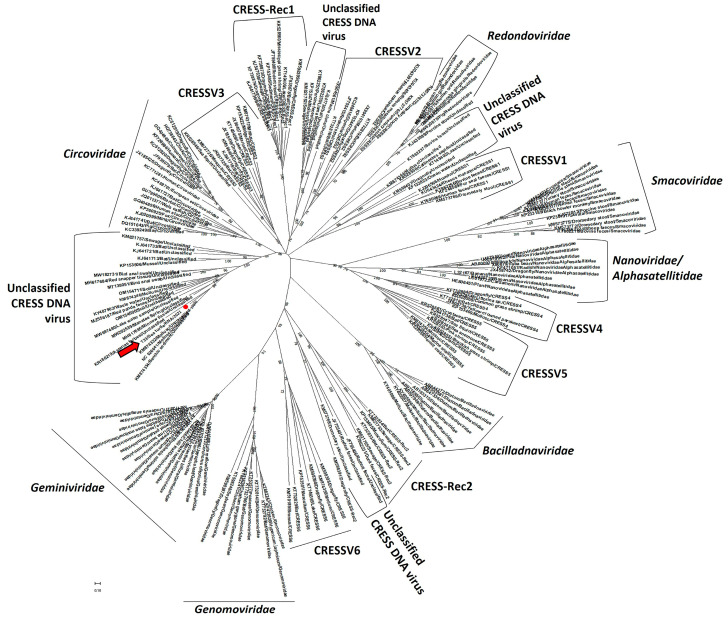
Phylogenetic analysis of the full-length deduced amino acid (aa) sequence of the putative replication initiation protein (Rep) of sea turtle-associated CRESS DNA virus T33 (highlighted with a red circle and indicated with a red arrow) with those of viruses representing the seven established viral families, six well-defined groups of unclassified CRESS DNA viruses (CRESSV1-6), two conserved groups of chimeric Reps (CRESS-Rec1 and -Rec2), and other unclassified CRESS DNA viruses within phylum *Cressdnaviricota* [18,19]. The virus name/source (detected in animal species)/country/year of sampling is shown for CRESS DNA virus T33, whilst the GenBank accession number/source (detected in animal species or environment)/classification status within phylum *Cressdnaviricota* have been mentioned for the other CRESS DNA viruses. Scale bar, 0.1 substitutions per aa residue. Bootstrap values of <60 are not shown. An enlarged image of the phylogenetic tree is shown in Supplementary Figure S4.

The putative Rep of sea turtle-associated CRESS DNA virus T33 was 322 aa in size and retained the three putative motifs (motif-I, -II and -III) of the HUH (Rolling Cycle Replication (RCR)) endonuclease domain and the three putative motifs (Walker-A, -B, and motif-C) of the SF3H domain that are conserved in CRESS DNA viruses [18,22] (Figure 4). The putative S3H domain of T33 Rep also retained the “arginine finger” motif that is conserved in *Bacilladnaviridae*, *Circoviridae*, *Nanoviridae*, *Smacoviridae*, and CRESSV1-5, but is absent in *Geminiviridae*, *Genomoviridae,* and CRESSV6 [18] (Figure 4). The T33 Rep shared a maximum pairwise deduced aa identity of 57.74% with that of an unclassified CRESS DNA virus detected from benthic sediment (GenBank accession number KM874334), followed by identities of 57.23% and 56.91% with mollusk-associated unclassified CRESS DNA viruses (KM874332 and NC_026641, respectively) [23] (Supplementary Figure S2). By phylogenetic analysis, the putative Rep of T33 formed a distinct branch with the CRESS DNA viruses from benthic sediment and mollusks (KM874334, KM874332, and NC_026641, respectively) as neighbors, within an isolated cluster of unclassified CRESS DNA viruses (Figure 5), corroborating observations based on deduced aa identities. Among the established CRESS DNA viral families and groups, the *Circoviridae* clade was located nearest to the cluster consisting of T33 and other unclassified CRESS DNA viruses (Figure 5). The putative Cap (370 aa in size) of T33 shared a maximum pairwise deduced aa identity of 30.51% with an unclassified CRESS DNA virus from a capybara (GenBank accession number MK570169) (Supplementary Figure S3). However, the T33 Cap lacked the characteristic arginine rich region in the N terminus.

To our knowledge, this is the first report on the detection of CRESS DNA viruses from sea turtles, adding yet another animal species to the rapidly expanding and diverse range of living forms from which these viruses have been reported, especially in marine life where information is lacking compared with terrestrial species [18,22,23,24,25,26,27,28]. A complete genome analysis of sea turtle-associated CRESS DNA virus T33 identified a novel, unclassified CRESS DNA virus, providing insights into the high genetic diversity between viruses within the phylum *Cressdnaviricota* [18,22]. Sea turtle T3 was sampled on the beach shortly after being captured from ocean waters, and the partial Rep sequence of virus T3 was more related (~75% identities) to that of a CRESS DNA virus (classified under *Circoviridae*) from a mollusk than those of other viruses [28]. These observations indicated that sea turtle T3 might have acquired the virus in an oceanic environment, although analysis of the complete genome, or at least the full-length Rep, would be required to infer the origin of sea turtle-associated CRESS DNA virus T3. On the other hand, since T33 was sampled before release from rehabilitation, we could not determine whether the virus was acquired during rehabilitation or in the ocean. However, sequence identities and phylogenetic analysis of the full-length putative Rep sequences identified unclassified CRESS DNA viruses from benthic sediment and mollusks [23] as the nearest relatives to T33, indicating a potential marine origin of sea turtle-associated CRESS DNA virus T33. Except for a blood sample from T33 that tested negative for CRESS DNA viruses, other tissue samples were not available from the sea turtles. Therefore, we could not establish whether viral strains T3 and T33 infected the sea turtles or were of dietary origin. The possibility of a dietary origin of the sea turtle-associated CRESS DNA viruses cannot be ruled out, especially as sea turtles feed on mollusks, and both T3 and T33 were more related (although shared low sequence identities) to mollusk-associated CRESS DNA viruses than other viruses.

Although the inclusion of sea turtles in the IUCN red list has created awareness on the roles of environmental pollution and climate change in the decline of sea turtle populations, to date, there is a lack of knowledge on the implications of infectious agents, especially viruses, on sea turtle morbidity and mortality, and on marine biodiversity impacting the habitats of sea turtles [4,5]. Furthermore, sea turtles can facilitate interspecies transmission of viruses between marine and terrestrial animals, especially when they visit coastal lands during nesting [4]. Since 1955, thirty-six episodes of mass mortality events (MME) involving 18 species of marine animals have been documented, and viruses were identified as the main etiological agents triggering these MME epizootics [42]. Considering these observations, marine life conservationists and one-health researchers should prioritize studies on virus discovery, surveillance, and pathogenesis in sea turtles.

## Data Availability

Additional data are available in Supplementary Table S1 and Figures S2–S4.

## Supplementary Materials

The following supporting information can be downloaded at: https://www.mdpi.com/article/10.3390/pathogens12040601/s1, Supplementary Table S1: Details of the cloacal samples obtained from sea turtles in coastal waters around the Caribbean Islands of St. Kitts and Nevis. The samples that tested positive for CRESS DNA viruses are highlighted in yellow; Supplementary Figure S2: Two-dimensional graphical representation of pairwise deduced amino acid sequence (%) identities between the putative capsid proteins (Rep) of CRESS DNA viruses within the phylum *Cressdnaviricota*. The plot was constructed using the Sequence Demarcation Tool Version 1.2 (SDTv1.2) with the MUSCLE alignment algorithm, as described previously [31,32,34,35]. The sea turtle-associated CRESS DNA virus T33 is highlighted with a red arrow; Supplementary Figure S3: Two-dimensional graphical representation of pairwise deduced amino acid sequence (%) identities between the putative capsid proteins (Cap) of CRESS DNA viruses. The plot was constructed using the Sequence Demarcation Tool Version 1.2 (SDTv1.2) with the MUSCLE alignment algorithm, as described previously [31,32,34,35]. The sea turtle-associated CRESS DNA virus T33 is highlighted with a red arrow; Supplementary Figure S4: Enlarged image of Figure 5.
