# Supplementary material for: First Report on Detection and Complete Genomic Analysis of a Novel CRESS DNA Virus from Sea Turtles
Source: Pathogens. 2023 Apr 15;12(4):601. doi: 10.3390/pathogens12040601 (PMC10142553; doi:10.3390/pathogens12040601)
Supplement: Supplementary file 1 [file pathogens-12-00601-s001.zip › Supplementary Table S1.pdf]

Supplementary Table S1. Details of the cloacal samples obtained from sea turtles in coastal waters around the Caribbean Islands of St. Kitts and Nevis. The samples that tested positive for CRESS DNA viruses are highlighted with yellow.

Sample/sea turtle number	Sea turtle species	Sampling [1]/Rescue [2] location	Month, year of collection	Lesions/Presence of epibiota
T1 [3]	Green	South Friars Beach [1]	July, 2021	Epibiota
T2	Green	Whitehouse Bay [1]	December, 2021	Cutaneous lesions
T3 [4]	Hawksbill	Whitehouse Bay [1]	November, 2021	Epibiota
T4	Green	South Friars Beach [1]	November, 2021	Epibiota
T5	Green	Whitehouse Bay [1]	February, 2022	Epibiota
T6	Hawksbill	South Friars Beach [1]	January, 2022	Epibiota
T7	Hawksbill	Whitehouse Bay [1]	November, 2021	Epibiota
T8	Green	South Friars Beach [1]	January, 2022	Epibiota
T9	Green	Whitehouse Bay [1]	November, 2021	Epibiota
T10	Green	South Friars Beach [1]	November, 2021	Epibiota
T11	Green	Shitten Bay [1]	October, 2021	Epibiota
T12 [5]	Green	South Friars Beach [1]	September, 2021	Epibiota
T13	Green	South Friars Beach [1]	October, 2021	Epibiota
T14	Green	South Friars Beach [1]	October, 2021	Epibiota
T15	Hawksbill	Shitten bay [1]	February, 2022	Epibiota
T16	Green	Shitten bay [1]	February, 2022	Epibiota
T17	Hawksbill	Timothy Beach [1]	March, 2022	Epibiota
T18	Green	South Friars Beach [1]	April, 2022	Epibiota
T19 [3]	Green	South Friars Beach [1]	April, 2022	Epibiota
T20 [4]	Hawksbill	Whitehouse Bay [1]	April, 2022	Epibiota
T21 [5]	Green	South Friars Beach [1]	April, 2022	Cutaneous lesions
T22	Green	South Friars Beach [1]	April, 2022	Epibiota
T23	Green	South Friars Beach [1]	April, 2022	Epibiota
T24	Hawksbill	South Friars Beach [1]	April, 2022	Epibiota
T25	Hawksbill	South Friars Beach [1]	April, 2022	Epibiota
T26	Green	South Friars Beach [1]	July, 2022	Epibiota
T27	Green	Whitehouse Bay [1]	June, 2022	Epibiota
T28	Green	South Friars Beach [1]	May, 2022	Epibiota
T29	Green	South Friars Beach [1]	August, 2022	Epibiota
T30	Green	South Friars Beach [1]	May, 2022	Epibiota
T31	Hawksbill	Whitehouse Bay [1]	July, 2022	Epibiota
T32 [6]	Loggerhead	Caribbean Sea [2, 6]	June, 2021 [6]	Boat strike injury
T33 [7]	Loggerhead	New River Ghaut [2, 7]	June, 2021 [7]	Flipper injury
T34 [8]	Hawksbill	South Friars Beach [2, 8]	June, 2021 [8]	Hook injury

[1] Sampling location
[2] Rescue location
[3-5] Sea turtles sampled twice during the study period: T1 [3] and T19 [3], T3 [4] and T20 [4], and T12 [5] and T21 [5].
[6] Sea turtle T32 was struck by a boat in the Caribbean Sea surrounding St. Kitts and Nevis, rescued by a local fisherman, and rehabilitated by the St. Kitts Sea Turtle Monitoring Network (SKSTMN). The cloacal sample was collected in captivity during rehabilitation in June of 2021.
[7] Sea turtle T33 was stranded due to flipper injury at New River Ghaut in October of 2019. The sea turtle was sampled in captivity before release from rehabilitation by the SKSTMN in June of 2021.
[8] Sea turtle T34 was found on the South Friars Beach and presented for removal of a hook. The cloacal sample was collected in captivity during rehabilitation by the SKSTMN in June of 2021.
