# Supplementary figures and images for: First Report on Detection and Complete Genomic Analysis of a Novel CRESS DNA Virus from Sea Turtles
Source: Pathogens. 2023 Apr 15;12(4):601. doi: 10.3390/pathogens12040601 (PMC10142553; doi:10.3390/pathogens12040601)

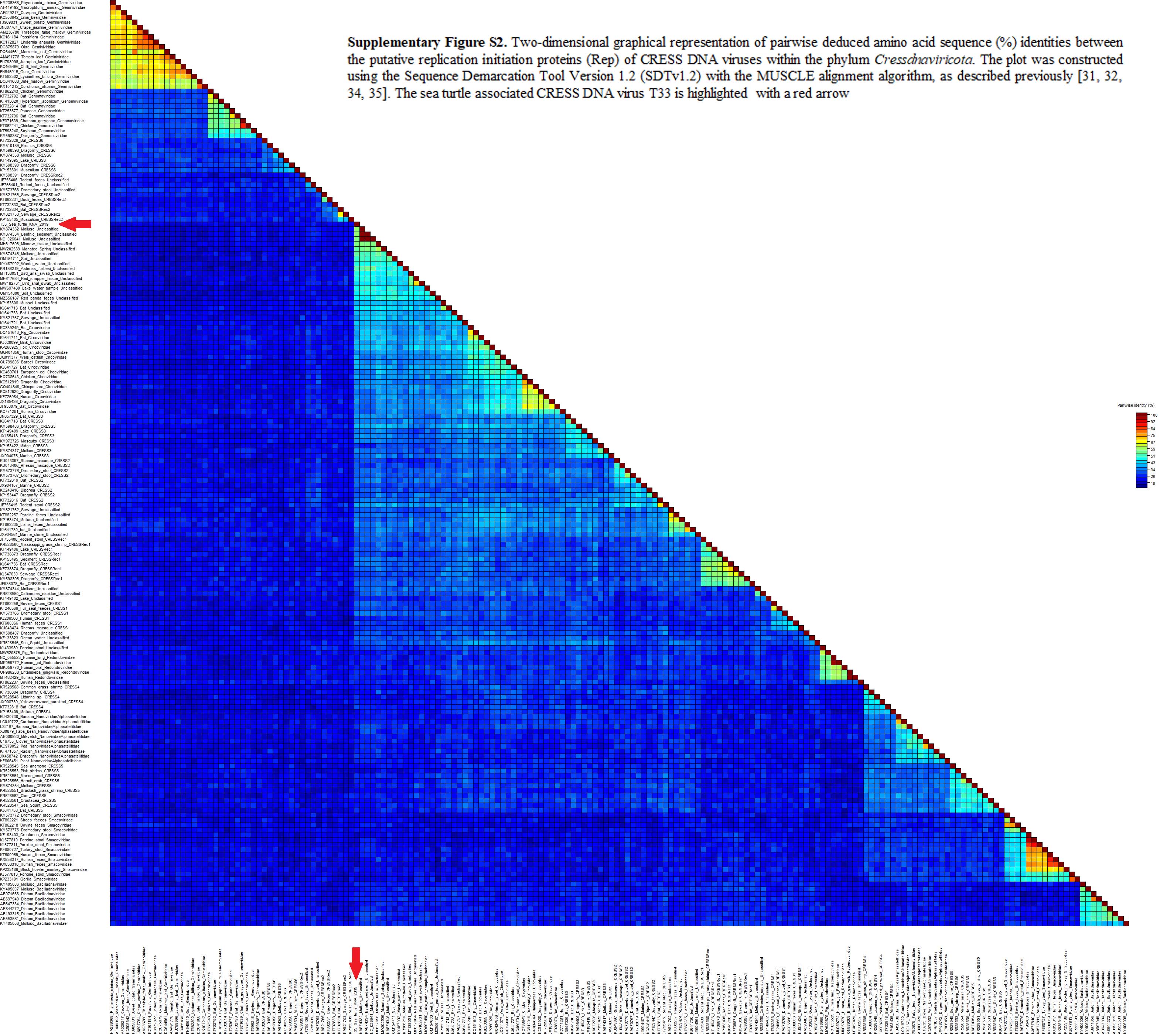

Supplement: Supplementary file 1 [file pathogens-12-00601-s001.zip › 2_Supplemetary Figure S2.jpg]

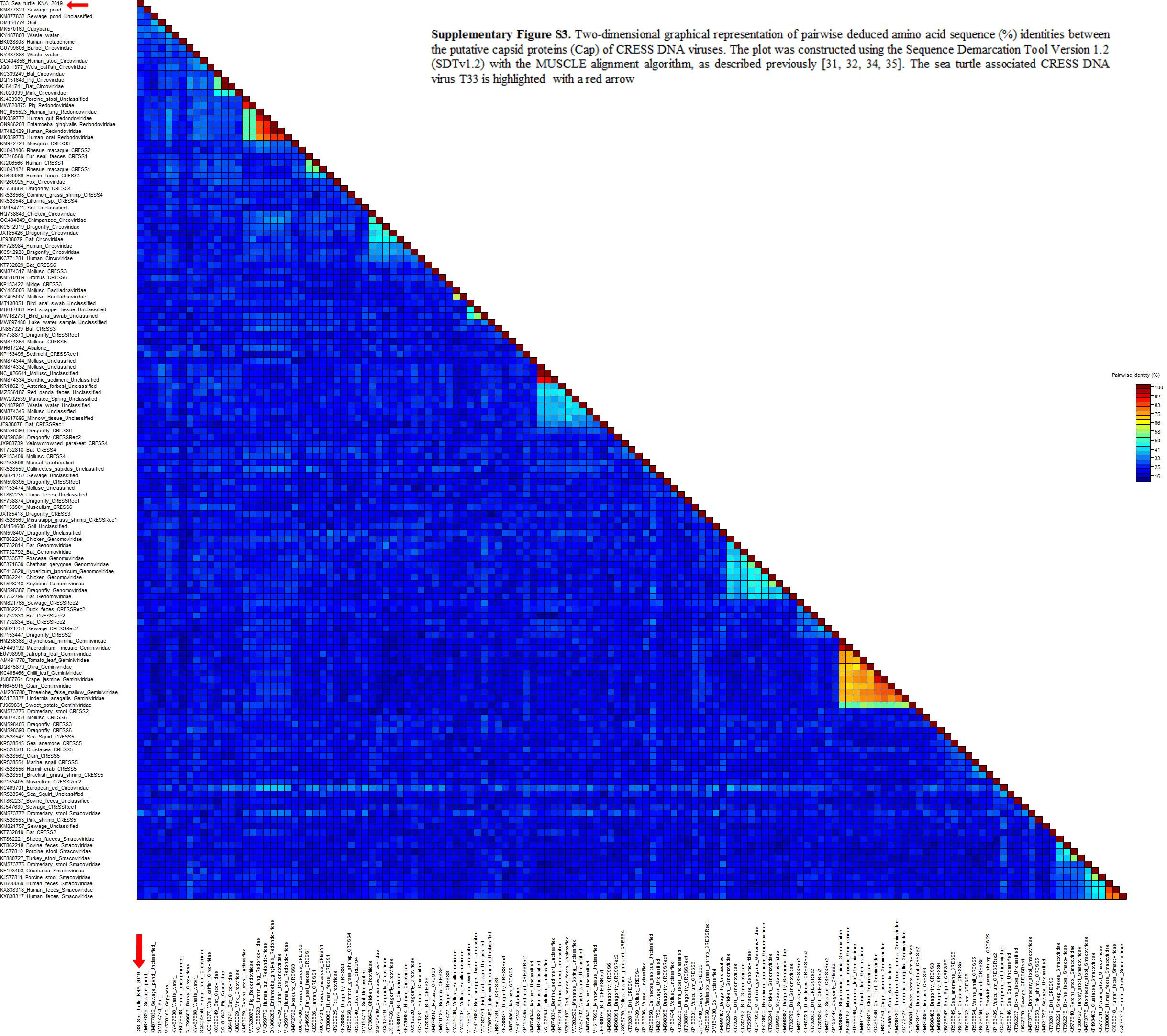

Supplement: Supplementary file 1 [file pathogens-12-00601-s001.zip › 3_Supplementary Figure S3.jpg]

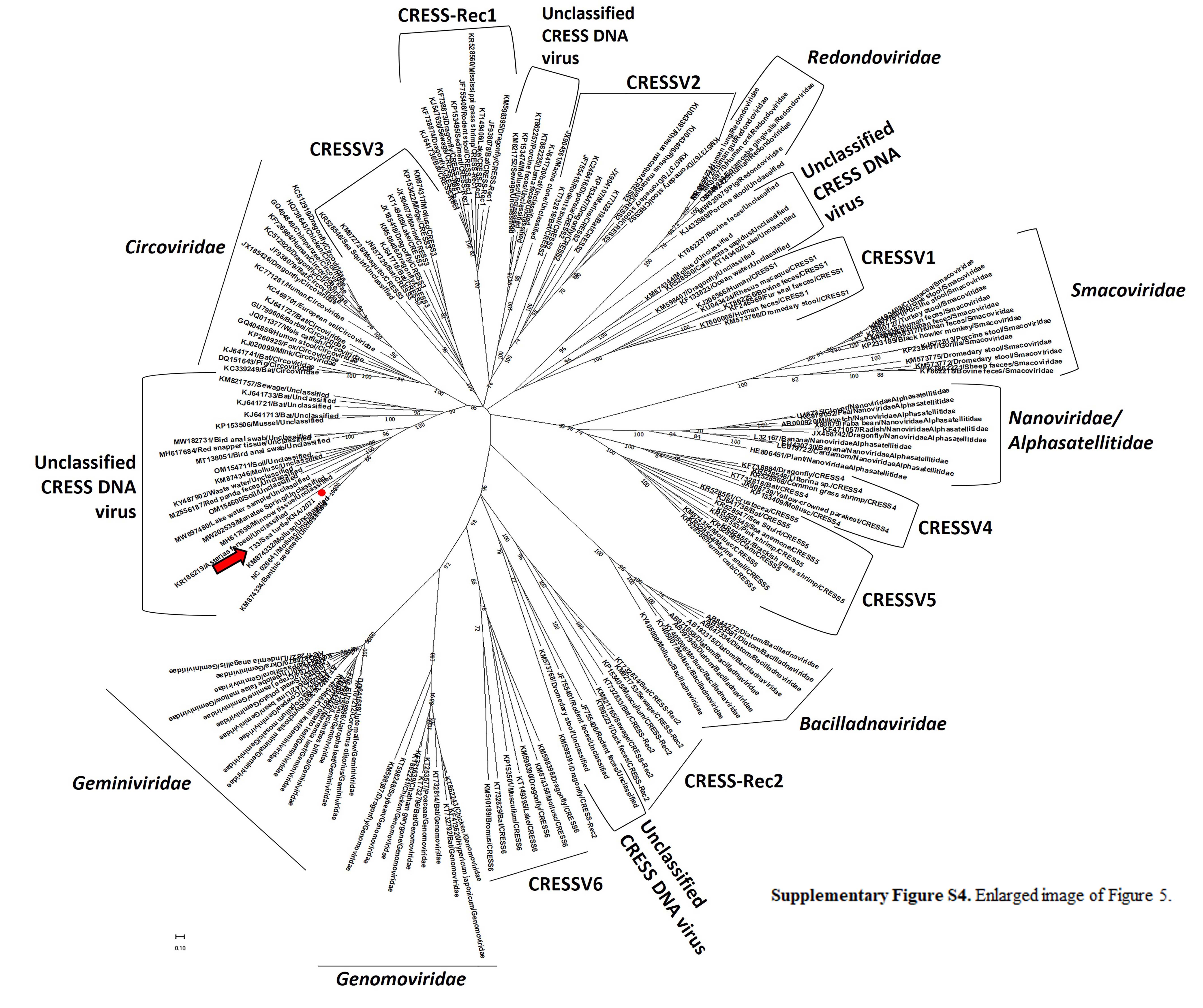

Supplement: Supplementary file 1 [file pathogens-12-00601-s001.zip › 4_Supplementary Figure S4.jpg]
